# Integrative Morphometric and Molecular Approach to Update the Impact and Distribution of Potato Cyst Nematodes *Globodera rostochiensis* and *Globodera pallida* (Tylenchida: Heteroderidae) in Algeria

**DOI:** 10.3390/pathogens10020216

**Published:** 2021-02-16

**Authors:** Aouicha Djebroune, Gahdab Chakali, Eugénia de Andrade, Maria João Camacho, Leidy Rusinque, Maria L. Inácio

**Affiliations:** 1 Département des Sciences Agronomiques, Faculté des Sciences de la Nature et de la Vie et des Sciences de la Terre, Université Djilali Bounaama Khemis Miliana, Route de Theniet El Had, 44225 Ain Defla, Algeria; djebrouneaouicha@hotmail.fr; 2 Département de Zoologie Agricole et Forestière, Ecole Nationale Supérieure Agronomique, El-Harrach, 16200 Algiers, Algeria; chakali_gahdab@yahoo.fr; 3 Instituto Nacional de Investigação Agrária e Veterinária (INIAV, I.P.), Quinta do Marquês, 2780-159 Oeiras, Portugal; eugenia.andrade@iniav.pt (E.d.A.); mjoao.camacho@iniav.pt (M.J.C.); leidy.rusinque@iniav.pt (L.R.); 4 GREEN-IT Bioresources for Sustainability, ITQB NOVA, Av. da República, 2780-157 Oeiras, Portugal

**Keywords:** potato cyst nematodes, morphology, PCR, geographical distribution, Algeria

## Abstract

Morphological and molecular studies were conducted to characterize the specific identity of 36 isolates of potato cyst nematodes (PCNs) recovered from soil samples collected in several potato producing areas of Algeria. Morphometric data revealed that 44% of isolates contained *Globodera pallida* alone, 28% contained *Globodera rostochiensis* alone and 28% mixtures of the two species. Morphometric values of cysts and second-stage juveniles were generally distributed with slight differences in the expected ranges for both *Globodera* species. Inter- and intraspecific morphometric variability in nematode isolates was noted. Molecular analysis using conventional multiplex PCR with species-specific primers and TaqMan real-time PCR confirmed the morphological identification. In addition, the distribution of both potato cyst nematode species throughout various parts of the country was investigated. In the central areas, the isolates of *G. pallida* alone dominate, whereas isolates of *G. rostochiensis* alone are more frequent in the southern areas. In the eastern regions, mixed isolates are more representative. Most isolates examined in the western areas are mixtures of the two species or *G. rostochiensis* alone. Comparatively, *G. pallida* remains the most widely distributed species in its geographic range. This study confirms the presence of two PCN species, *G. pallida* and *G. rostochiensis*, in Algeria and provides additional information on their biogeographic distribution.

## 1. Introduction

Potato cyst nematodes (PCNs), *Globodera rostochiensis* [1,2] and *Globodera pallida* [3], are damaging to potato (*Solanum tuberosum* L.) in various countries [4]. These parasites constitute the second group of the 10 main plant-parasitic nematodes of scientific and economic importance [5], causing annual losses estimated at 9% of world potato production [6]. Due to their harmful potential, both species are classified as quarantine organisms and were added to the European and Mediterranean Plant Protection Organization (EPPO) A2 list in 1975 [7]. These nematode species originated in the Andean region of South America [8] and from there they have spread to different parts of the world, mainly by soils adhering to potato tubers from infested fields. PCN have been reported throughout Europe, South America and parts of Asia, North America, Oceania and Africa where potatoes are grown [7]. However, new detections of *Globodera* sp. continue to be reported [9,10,11,12,13,14,15].

The emergence of potato cyst nematodes in Algeria was noted in 1953 at a few fields in the Algiers region. Their introduction dates back to the 1940s with potato seed imported from England [16], soon after the II World War. Since then, these parasitic nematodes have taken a large extension in the national territory [17]. Recent data on the evolution of these parasites in some regions of the centre and south of the country confirmed the presence of the two *Globodera* species [18,19]. However, in what concerns their pest status categorization, both species are considered present in the country but with restricted distribution [20].

Currently, potato cultivation has a larger extension in various areas of Algeria where El-Oued, Ain-Defla, Mostaganem, Mascara and Bouira hold the largest production areas [21]. Three annual types of production are conducted: the seasonal crop (planting January–March) which is practiced in all regions of the country, the last-season crop (planting July–August) which occupies second place and is limited to irrigable areas in summer and the early crop (planting October–November) which occupies a limited place on the coast at mild temperatures [22]. Data of 2017 revealed an area of 148,822 hectares of potato cultivated to ensure a production of 4.606 million tonnes [21]. This enables us to classify Algeria at the first rank in potato production in Africa [23].

Given the considerable economic losses caused by these nematodes, the characterization and evolution of the two PCN species in their respective biotopes has become fundamental. In this regard, the morphometric criteria applicable to the perineal region of cysts and second-stage juveniles (J2) are considered to be essential elements for differential diagnosis of the two species of PCN [15,24,25,26,27,28,29,30,31,32,33,34,35,36,37,38,39]. However, the great morphological and morphometric similarities and the overlap of various diagnostic characters between these two species often lead to confusion [35,40]. Molecular analyses based on DNA examination prove essential for more reliable differentiation between PCN species. Various tests are successfully developed and applied, of which the polymerase chain reaction (PCR) is the method of choice. Several PCR-based strategies were found to be useful to differentiate *G. pallida* from *G. rostochiensis*, including conventional PCR with species-specific primers used in single or multiplex reactions [28,41,42,43] and real-time PCR using double-strand binding dyes such as SYBRGreen or hybridization probes such as TaqMan [33,44,45,46].

In this study, a characterization of PCN isolates was conducted in their biotope throughout the potato producing areas of Algeria. A morphometric analysis validated by both molecular approaches, conventional multiplex PCR with species-specific primers and real-time PCR with TaqMan probes, were conducted for a specific identification. The biogeography of the two nematode species is essential information to best manage the alternatives to undertake as part of the protection of the potato crop.

## 2. Results

### 2.1. Morphological and Morphometric Analysis

#### 2.1.1. Descriptive Characters

**Figure 1:**
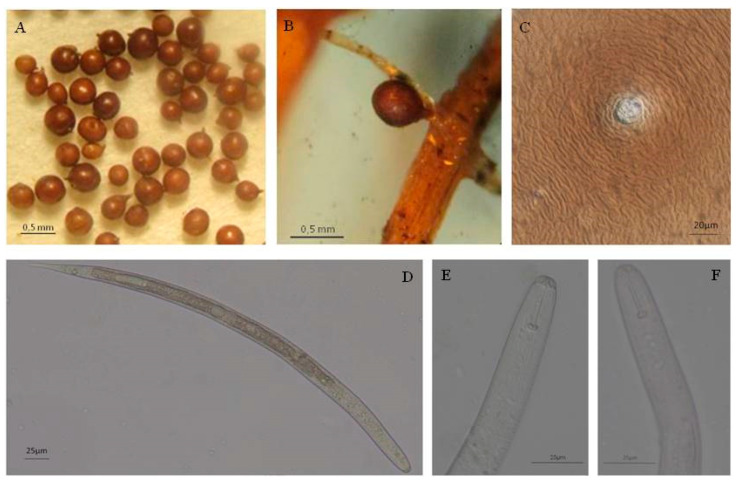
Cyst nematodes associated with potato: (**A**), cysts; (**B**), cyst attached to the root; (**C**), perineal region of *Globodera pallida*; (**D**), juvenile J2; (**E**), pointed basal knobs of *G. pallida*; (**F**), rounded basal knobs of *G. rostochiensis*.

Specimens of Algerian isolates of potato cyst nematodes are described below:

Cysts: Cysts were rounded to globular in shape with a protruding neck and light brown to dark brown in colour (Figure 1A). By its shape, the neck facilitated the attachment of cysts to the root of its host (Figure 1B). The perineal regions had two spaced openings of different size; the larger one represented the vulva fenestrate and the second the reduced anus with a V-shaped mark. Characteristic circular ridges were located between the fenestra and the anus (Figure 1C).

Second-stage juveniles (J2): They were vermiform, and tap at the tail with a hyaline part; ventro-lateral overlapping of esophageal glands over intestine (Figure 1D). The head was rounded and slightly offset with prominent cephalic sclerotization. The mouth had an apparent stylet developed with pointed basal knobs in *Globodera pallida* (Figure 1E) and rounded in *G. rostochiensis* (Figure 1F).

#### 2.1.2. Morphometric Data

Morphometric investigation of cysts and J2s allowed a first specific identification of the different isolates of potato cyst nematodes. *Globodera pallida* and *G. rostochiensis* species were present separately or as a mixture in the various fields prospected.

##### *Globodera* *pallida*

*Globodera pallida* species was noted in 16 fields represented by 9 to 22, 26 and 29 isolates (Table 1 and Table 2). The morphometric of the specimens analyzed was comparable overall to that of *G. pallida* mentioned by [40,47]. The measurements and the mean values calculated were distributed with slight variations in the ranges proposed for this species. The majority of isolates showed a greater upper limit of the fenestra–anus distance than that defined for *G. pallida* (67 μm). The average values of the vulva diameter of the samples except for 9, 11 and 20 isolates were superior to the maximum value of *G. pallida* (21 µm). Most of the extreme values of the vulva diameter lay outside the measurement range for *G. pallida* (18–21 µm). The maximum stylet lengths for 9 and 17 isolates were respectively 27.2 and 27.6 μm, slightly exceeding the values reported for *G. pallida* (26 μm).

##### *Globodera* *rostochiensis*

A total of 10 *G. rostochiensis* isolates (3, 7, 23, 24, 25, 27, 28, 30, 32 and 34) (Table 1 and Table 2) were identified. The morphometric features of the cysts and juveniles studied correspond to those proposed for *G. rostochiensis* by [40,47]. Measurements and calculated averages lay with some slight differences in the ranges of *G. rostochiensis*. The mean values of the vulva-anus distance and vulva diameter of isolates 27, 28 and 30 from the Saharan region (El Oued) exceed the maximum values reported for *G. rostochiensis*. Likewise, the upper limits values of the vulva-anus distance of all isolates, except for 25 and 32 isolates, are higher than those expected for *G. rostochiensis* (77 µm). The upper limit of the vulva diameter of all isolates was above 20 µm, which was not very typical for the species. The maximum stylet length for 24, 27, 28 and 30 isolates slightly exceeded that reported for *G. rostochiensis* (23 µm).

##### Mixture of *Globodera pallida* and *G. rostochiensis*

The two *Globodera* species were present sympatrially in 10 fields represented by 1, 2, 4, 5, 6, 8, 31, 33, 35 and 36 isolates (Table 3 and Table 4). In each of the isolates, some specimens show the morphometric characteristics of *G. pallida* and others of *G. rostochiensis*. The measurements and average values were within the ranges of these both nematode species. However, the mean and extreme values of the vulva diameter of some isolates lay outside the ranges of PCN species. Similarly, the averages and maximum values of the vulva–anus distance of some isolates exceeded those proposed for *G. pallida* and *G. rostochiensis*.

**Figure 2:**
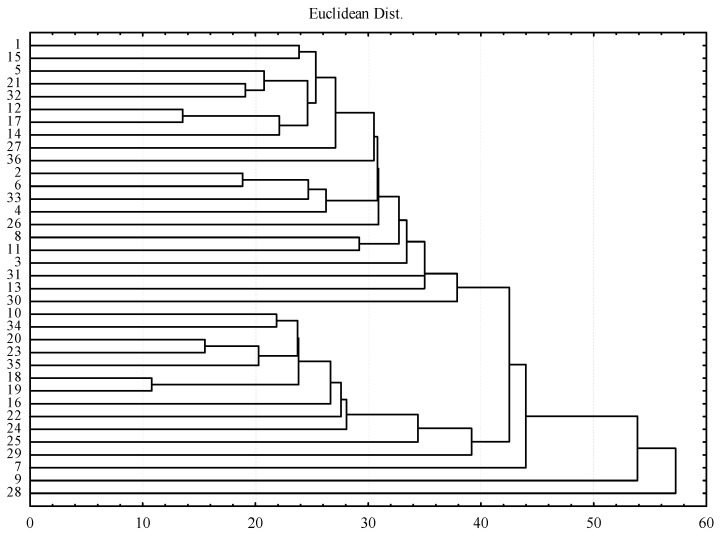
Hierarchical classification of the 36 Algerian isolates of potato cyst nematodes *Globodera* spp.

In addition, isolates 2, 6 and 31 showed a dominance of *G. pallida*, while *G. rostochiensis* was frequent in isolates 1, 4, 5, 8, 33, 35 and 36. The ascending hierarchical classification carried out on 17 morphometric characters of cysts and second stage juveniles (Figure 2) allowed separating the studied nematode isolates into five groups with different constitutive status, which further confirmed the morphometric variability of these isolates. The first two groups were respectively represented by isolates 1, 15, 5, 21, 32, 12, 17, 14, 27, 36, 2, 6, 33, 4, 26, 8, 11, 3, 31, 13 and 30 and isolates 10, 34, 20, 23, 35, 18, 19, 16, 22, 24, 25 and 29, belonging to various geographical origins and containing a single *Globodera* species or both species. This means that the morphometric similarity of the isolates of these nematodes was not related to the geographic origin of the isolate and the species it represented. The isolates 7, 9 and 28 were distributed distinctly between the third and fifth group.

The morphometric studies of specimens of the considered nematode isolates showed that among the cysts analyzed, 203 cysts were identified as *G. pallida* and 157 cysts represented *G. rostochiensis*. In the case of juveniles, a total of 207 individuals were found belonging to *G. pallida* and 153 individuals to *G. rostochiensis*. Average morphometric values of the cysts and J2s of each *Globodera* species are regrouped in Table 5.

The morphometric values obtained on all the characters overlapped between the two PCN species. Analysis of the average values showed a difference between these both nematode species. For the perineal regions of cysts, a substantial variation was recorded for fenestra to anus distance (56.45 ± 12.17 and 73.83 ± 21.29 μm for *G. pallida* and *G. rostochiensis*, respectively). Likewise, a difference was noted between the number of cuticular ridges between fenestra and anus (12.43 ± 3.04 μm for *G. pallida* and 18.33 ± 4.21 μm for *G. rostochiensis*), the vulva diameter (18.90 ± 3.89μm for *G. rostochiensis* and 21.82 ± 4.76 μm for *G. pallida*) and the Granek’s ratio (2.62 ± 0.48 and 4.00 ± 1.12 μm for *G. pallida* and *G. rostochiensis*, respectively). Regarding second stage juveniles, the results indicated that *G. rostochiensis* had a smaller body (432.99 ± 46.52 μm) than *G. pallida* (440.68 ± 28.71 μm). Additionally, the stylet showed a length of 24.07 ± 1.08 μm, with pointed basal knobs in *G. pallida* and a length of 22.20 ± 0.88 μm, with rounded basal knobs in *G. rostochiensis*. The tail was relatively short in *G. rostochiensis* (45.63 ± 4.78 μm) compared to *G. pallida* (47.46 ± 4.19 μm). Moreover, the comparison of the mean morphometric values revealed a significant difference (*p* < 0.05) between the isolates of both potato cyst nematode species for all characters considered on the biological material examined.

Variability between the mean morphometric values was also recorded between the isolates of each *Globodera* species. For *G. pallida* isolates, a significant difference (*p* < 0.05) was noted for all the characters except for vulva-anus distance (*p* = 0.0956), the vulva diameter (*p* = 0.0692) and juvenile body width in the middle (*p* = 0.5656). For *G. rostochiensis* isolates, all characters showed significance except for the Granek’s ratio (*p* = 0.1413). These results showed great inter- and intra-specific morphometric variability.

### 2.2. Molecular Identification

#### 2.2.1. Conventional Multiplex PCR

**Figure 3:**
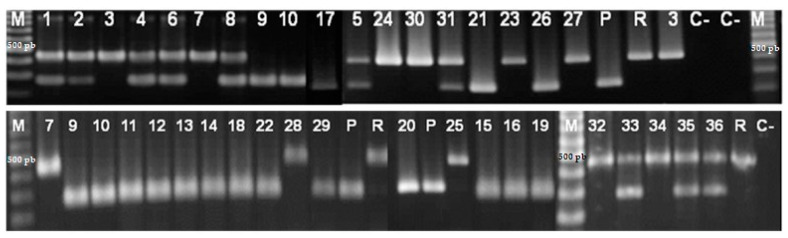
DNA amplification product (multiplex PCR) of potato cyst nematode isolates; 1–36: Algerian *Globodera* spp., M: DNA marker of size 100 bp., C-: molecular grade water negative control or distilled water negative control, *p*: *Globodera pallida* positive control, R: *G. rostochiensis* positive control.

All DNA extracts from the Algeria nematode samples produced fragments of the same size as those obtained from the *G. pallida* and *G. rostochiensis* positive controls, which confirmed that the multiplex PCR reactions proceeded correctly and allowed DNA amplification (Figure 3). A total of 16 samples (9 to 22, 26 and 29) yielded a single fragment of 265 bp, specific for *G. pallida*. Ten other samples (3, 7, 23, 24, 25, 27, 28, 30, 32 and 34) produced a single fragment of 434 bp, specific for *G. rostochiensis*. These two fragments occurred in the various samples (1, 2, 4, 5, 6, 8, 31, 33, 35 and 36) showing that these samples contained a mixture of both *Globodera* species. Therefore, multiplex PCR with species-specific primers confirmed the morphological identification of all tested PCN isolates.

No PCR product was obtained in the negative controls without DNA template, which means the absence of contamination during the PCR reaction preparation.

#### 2.2.2. TaqMan Real-Time PCR

The fluorescence of FAM (Flurescein amidites) and TET (Tetrachlorofluorescein) was obtained in the nematode DNA samples during TaqMan real-time PCR assay (Table 6). A total of 16 samples (9 to 22, 26 and 29) emitted only the fluorescence of FAM which corresponded to *Globodera pallida* and 10 samples yielded only the fluorescence of TET which corresponded to *G. rostochiensis.* Both types of fluorescence were noted in 10 samples (1, 2, 4, 5, 6, 8, 31, 33, 35 and 36), which indicated that these isolates contained the two PCN species. No fluorescence was observed in the negative controls. Therefore, the TaqMan real-time PCR results further confirmed the specific identity of the considered nematode isolates.

The mean threshold cycle (Ct) values highlighted a difference between DNA samples. For the detection of *G. pallida*, average Ct values varied from 14.25 to 29.19 which corresponded to DNA isolated from 17 and 29 isolates, respectively. Regarding the detection of *G. rostochiensis*, the mean Ct values were between 16.55 and 24.09 recorded respectively in the DNA extracted from 6 and 28 isolates.

**Figure 4:**
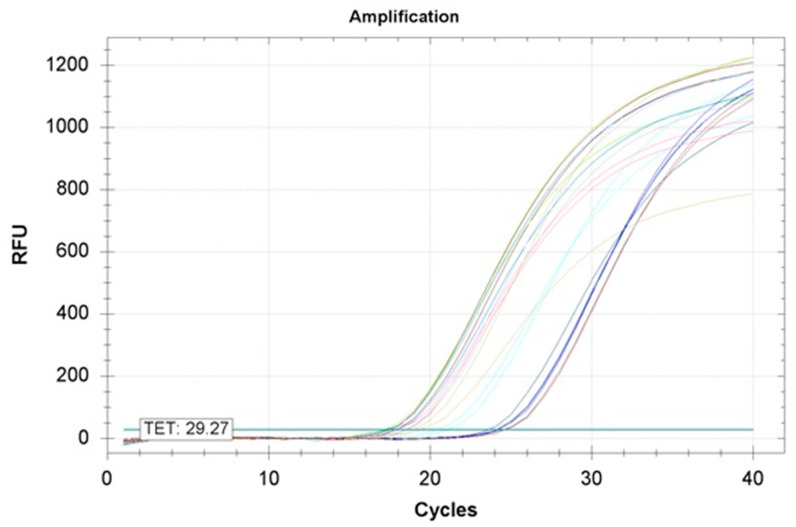
Some amplification curves of the ITS1 regions of rDNA from Algerian potato cyst nematode samples by TaqMan real-time PCR determining the amount of fluorescence as a function of the cycle numbers. (RFU: fluorescence. Two replicates of the same sample are represented by the same color).

Regarding the amplification curves in function of cycle numbers, all DNA samples showed typical amplification curves corresponding to a sigmoid shape (Figure 4).

### 2.3. Distribution of Potato Cyst Nematodes Species

**Figure 5:**
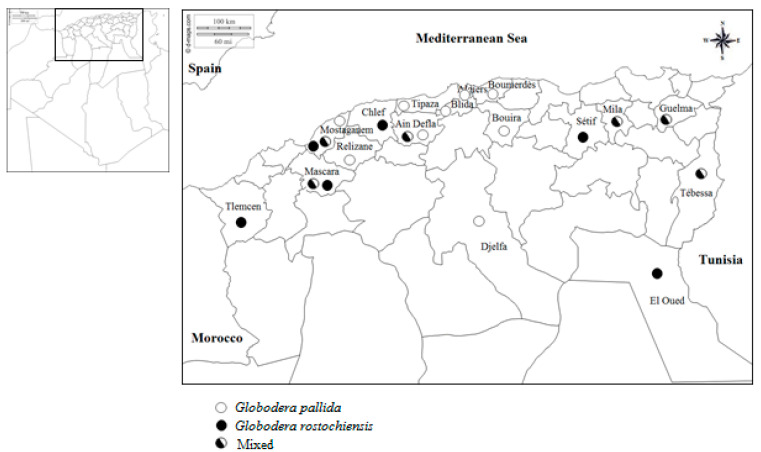
Biogeographic distribution of potato cyst nematodes *Globodera pallida* and *G. rostochiensis* in Algeria.

The nematode species *Globodera pallida* and *G. rostochiensis* had a wide spread in the potato producing regions of Algeria (Figure 5). Great variability in their distribution throughout the prospected range was noted. In central regions, *G. pallida* alone isolates dominated, while *G. rostochiensis* alone isolates were more frequent in southern regions. In the eastern areas, mixed isolates were more present. The majority of studied isolates in the western regions represented a mixture of the two species or *G. rostochiensis* alone isolates.

Some areas were infested by a single *Globodera* species and others by both species either separately or mixed in a population. All combinations were present.

In addition, the comparison of the species distribution showed that *G. pallida* had a more significant extension, as it was found in 13 regions out of the 17 prospected, while *G. rostochiensis* was present in only 10 regions.

## 3. Discussion

The morphological and morphometric data acquired on Algerian isolates of potato cyst nematodes are comparable to those provided by [40,47]. However, some slight differences are noted in the value amplitudes and the calculated means. This dissimilarity is due to the intra-specific variability for certain extreme values and remains very comparable with those reported by different authors in various geographic areas [26,31,33,34,35]. In various cases, obtained morphometric data has not been very conclusive in the differentiation between the nematode species *G. pallida* and *G. rostochiensis* and it is in this context that molecular analysis has been conducted for confirmation.

Conventional multiplex PCR assays with species-specific primers (ITS5/PITSp4 + PITSr3) and TaqMan real-time PCR produced consistent results and confirmation of the identity of the cyst nematode isolates associated with potato. Despite the consideration of the molecular tool for species characterization, the morphological approach remains basic in taxonomy.

The two *Globodera* species identified are found separately or mixed in the various fields surveyed. Examples of cases have been reported in the Ain Defla area by [18]. In this regard, only *G. pallida* or *G. rostochiensis* alone isolates in different regions (Algiers, Boumerdes, Blida, Tipaza, Béchar and El Oued) were identified by [19]. Similar investigations carried out in various other countries have shown the presence of alone isolates and mixed isolates of the PCN species [13,48,49,50,51,52], which corroborates with these results. In addition, according to [48], *G. pallida* and *G. rostochiensis* rarely occurred as separate species, but more often as a mixture species in the same field.

*Globodera pallida* alone isolates are present in 44% of the fields prospected, while *G. rostochiensis* alone isolates and mixed isolates are present in the rest of the fields with equal proportions (28%), showing the dominance of the *G. pallida* species in potato fields. This may result from the control of populations of *G. rostochiensis* through the intensive cultivation of resistant potato cultivars as is occurring in other countries like the Netherlands [53], United Kingdom [54,55,56] and Portugal [52] since almost all current potato cultivars are resistant or tolerant to this species [34], unlike with *G. pallida* species where the number of resistant varieties is limited [26,34,57,58].

The presence of mixed populations in fields represents a more worrying threat than pure populations for potato cultivation, not only because of yield losses, but also because of their extremely difficult management, especially when using resistant potato varieties as an alternative control method, since no cultivar is resistant to both *Globodera* species [59]. Repeated use of cultivars resistant to *G. rostochiensis* may favour the multiplication of *G. pallida* in mixed populations, which is the case in Ile de Ré [60] and in The Netherlands [61]. The consequence of the fusion is the possible reinforcement of a cross hybridization between these two nematode species that might result in a generation with new genotypes [34]. However, the crossing between these two species probably results in non-fertile hybrids [62].

Analysis of the data shows a wide geographical distribution of *Globodera* species in the regions prospected and with certain dominance. The majority of the isolates present in the central regions belong to the *G. pallida* species, while *G. rostochiensis* isolates are more frequent in the southern areas. In eastern regions, the two PCN species are often present in mixed isolates. Most of the samples identified in the western regions are mixed or *G. rostochiensis* alone. On this subject, it was reported by [19] that *G. pallida* mainly occupies northern Algeria, while *G. rostochiensis* occurs mainly in southern regions. The study conducted by [63] on the distribution of potato cyst nematode species in South America showed that north of 15.6° S, only the *G. pallida* species is noted, but south of this latitude, most of the examined populations belong to the species *G. rostochiensis* or to both common species. Based on these data, the distribution of both PCN species is related to latitude, especially the influence of day length. The predominance of *G. rostochiensis* in some regions in Tunisia is determined by temperature [27]. In this regard, according to [64] *G. rostochiensis* is more competitive than *G. pallida* at an average temperature of 24 °C and conversely at an average temperature of 9.5 °C. Recently, it was shown by [65,66] that the optimum temperature for reproduction of *G. rostochiensis* is higher than that of *G. pallida*. The distribution of *Globodera* species depends on a set of environmental variables that govern the nematode populations in their range.

The widespread distribution of nematodes of the genus *Globodera* in the potato-producing regions may be due to the environmental conditions favorable to the development of these pests. Various reports have shown that temperature is a factor limiting the development of these nematode species. In addition, the type of soil plays a considerable role in the development of these nematodes. It was noted that light and porous soils favor nematodes [67]. Added to this are the invoices linked to the host plant, particularly the varieties of potato cultivated (Table 7); Spunta and Desiree, which are preferential hosts for *G. pallida* and *G. rostochiensis,* are indicate by various authors [68,69,70,71,72,73]. In addition, human activities allow more ground movement and are a factor favouring the spread of nematode cysts.

Further PCN surveys should cover other areas with attention to the seed multiplication plots. In addition to the essential species identification, investigations into the detection of pathotypes within each species deserve to be conducted. Faced with the lack of information on varietal resistance, tests of susceptibility to nematodes must be carried out for a better knowledge of resistant varieties in order to better manage these harmful pests.

The control of PCN should be based on a combination of practices, such as the use of natural crop production methods like the resistant cultivars, rotations, and biological control agents. The starting point to guide decisions is to know about the presence and spread of both *Globodera* species. Thus, this study is a valuable contribution to have a more complete and updated picture of the distribution of PCN in Algeria.

## 4. Materials and Methods

### 4.1. Globodera *spp.* Collection and Isolation

Soil sampling was conducted between 2014 and 2018 in potato fields from 17 potato growing areas of Algeria. After the potato harvest, a 1-hectare sampling was considered in each field, where 60 subsamples were taken at a depth of 10–30 cm along both diagonals. The soil cores were mixed in a plastic bag to form a representative composite sample. In the laboratory, the soil was mixed and air dried, then 1 kg was retained for the extraction of cysts using the Fenwick method [74]. The cysts retrieved from each soil sample were collected separately in Eppendorf tubes and stored at room temperature. A total of 36 isolates of *Globodera* spp. were selected for analysis (Table 7).

### 4.2. Morphological and Morphometric Characterization

Morphometric analysis was carried out on 10 cysts and 10 juveniles J2 taken from each isolate collected. Each batch of juveniles J2 analyzed was extracted from these same cysts. The perineal region of the cysts was carefully cut under a stereoscope LEICA MZ6 with an ophthalmic scalpel. Subsequently, the perineal regions and the juveniles were mounted separately in distilled water on glass slides and examined using an Olympus BX-41 light microscope. The ProgResSpeed XT core 5—Jenoptik image software was used for the measurements and taking the pictures. A total of 13 morphometric criteria were measured (Table 1, Table 2, Table 3 and Table 4). Nematological indexes; a (body length/body width in the middle), c (body length/tail length) and c′ (tail length/body width at anus level) were calculated. Morphological identification of PCN species was carried out by combining the cysts and juveniles characters (fenestra diameter, fenestra to anus distance, number of cuticular ridges between fenestra and anus, Granek’s ratio: fenestra-anus distance divided by fenestra diameter; stylet length and stylet knobs shape) according to the identification keys proposed by [40,47]. In addition to the morphometric characters, the shape and colour of the cysts and the morphology of J2 were noted in order to further characterize the isolates analyzed.

### 4.3. Molecular Characterization

#### 4.3.1. DNA Extraction

Total DNA was extracted from 20 cysts of each isolate according to the method described by [41]. Cysts were crushed in a 1.5 mL microtube using a sterile plastic micro-pestle with 200 μL of extraction buffer containing 5 M guanidine isothiocyanate, 10 mM EDTA, 50 mM Tris-HCl (pH 7.5) supplemented with 8% of mercaptoethanol which was added after the crushing. The mixture was incubated at room temperature for 1 h without shaking. Equal volumes (200 μL) of phenol and chloroform:isoamyl alcohol (24:1) were added, and phases were separated by centrifugation at 13,000 rpm for 10 min. The supernatant was transferred to a new tube and DNA was cleaned up again by adding an equal volume of chloroform:isoamyl alcohol (24:1) followed by a centrifugation step at 13,000 rpm for 10 min. The DNA contained in the supernatant was precipitated by adding 200 µL of 0.3 M sodium acetate and 2 volumes of ethanol and centrifuged at 13,000 rpm for 10 min. The obtained DNA was air dried and dissolved in 100 μL of TE buffer. Finally, the DNA was evaluated quantitatively and qualitatively using a thermo-NANODROPP 2000 and stored at −20 °C until processing.

#### 4.3.2. Conventional Multiplex PCR with Specific Primers

The detection of both species of *Globodera* was performed by duplex conventional PCR. The internal transcribed spacer (ITS) region of the nematode ribosomal DNA (rDNA) was amplified using species-specific primers: PITSr3 (5′-AGCGCAGACATGCCGCAA-3′) for *G. rostochiensis* and PITSp4 (5′-ACAACAGCAATCGTCGAG-3′) for *G. pallida* in combination with common primer ITS5 (5′-GGAAGTAAAAGTCGTAACAAGG-3′) [41]. Multiplex PCR reactions were performed in a 25 μL total volume containing 5 μL template DNA (10 ng/µL), 5 μL 5X Green GoTaq Flexi buffer, 2 µM MgCl_2_ (25 mM), 0.40 µL each dNTP (10 mM), 0.63 µL each primer (10 µM), 0.5 µL GoTaq G2 Flexi DNA polymerase (5 U/µL) (Promega, Madison, CT, USA) and 10.23 μL of molecular grade water (MGW). The amplification was carried out in a thermocycler according to the procedure: an initial denaturation step of 94 °C for 2 min; followed by 35 cycles of 94 °C for 30 s, 55 °C for 30 s and 72 °C for 30 s and a final extension cycle of 72 °C for 7 min [47]. Positive controls for both *Globodera* species were taken into account in the amplifications for comparative and also two negative controls (blanks) with distilled water and molecular grade water (without DNA) to ensure that no environmental contamination occurred.

Amplified PCR products (5 µL of each reaction) were separated by electrophoresis on 1.5% agarose gel in 1× tris-acetate-EDTA (TAE) buffer. The gel was stained with GelRed Nucleic Acid Gel Stain (Biotium, Fremont, CA, USA) (150 μL of GelRed, 50 mL of NaCl 1M, 450 mL of H_2_0) for 10 min, then visualized and photographed under ultraviolet light. The expected size of amplicons is 265 base pair (bp) and 434 bp for *G. pallida* and *G. rostochiensis*, respectively.

#### 4.3.3. TaqMan Real-Time PCR

The detection of both pathogens was also performed by duplex qPCR. This test was carried out according to the protocol proposed by [47], aimed at amplifying the internal transcribed spacer 1 (ITS-1) region of rDNA. Two primers were considered: forward primer Glob 531F (5′-TGT-AGG-CTG-CTA-YTC-CAT-GTY-GT-3′) and reverse primer Glob 601R (5′-CCA-CGG-ACG-TAG-CACACA-AG-3′); and the two probes, one for *G. pallida* GP LNA (5′-TGCCGT-ACC-(C)(A)G-CGG-CAT-3′) labelled with the reporter dye FAM and the quencher BHQ-1 and the second for *G. rostochiensis* GR LNA (5′-GCC-GTA-CC(T)-(T)GC-GGC-AT-3′) labelled with the reporter dye TET and the quencher BHQ-1. The qPCR reactions were done in a final reaction volume of 20 μL composed of 3 μL nematode DNA extract (10 ng/µL), 10 μL of Maxima SYBR Green/ROX qPCR Master Mix (2×) (ThermoFisher Scientific, Waltham, MA, USA), 0.38 μL each primer (10 µM), 0.5 μL probe GP LNA (10 µM) and 1 μL probe GR LNA (10 µM). The remaining volume was filled with 4.75 μL of molecular grade water. The amplifications were carried out using a real-time PCR thermocycler (BioRad Laboratories, Hercules, CA, USA) in a plate covered with an adhesive film “Microseal”. The thermal cycling profile consisted of a uracil-DNA glycosylase (UDG) treatment for 2 min at 50 °C. This was followed by denaturation and polymerase activation at 95 °C for 10 min and 40 cycles of denaturation at 95 °C for 15 s and annealing-extension at 60 °C for 1 minute. All samples were analysed in two replicates. Negative controls (blanks) containing distilled water and molecular grade water (no DNA template) were included in the reaction.

The fluorescence emitted by the hydrolysis probes was measured after extension in all cycles. The analysis was performed by the Sequence Detection Software (Applied Biosystems, Foster City, CA, USA). Threshold value was set manually and baseline was set in an automatic mode.

Positive samples were those with an amplification curve for the corresponding fluorophore with a sigmoid shape. In the same way, negative samples were those lacking amplification with the specific fluorophores or with an atypical amplification curve.

#### 4.3.4. Data Analysis

An ascending hierarchical classification using the method of minimum skipping of the morphometric criteria of cysts and J2 was carried out to highlight the probable characteristics of the Globodera isolates collected in the areas prospected. Likewise, these morphometric characters underwent an analysis of variance (ANOVA) to test the significance of the mean values between the isolates of each Globodera species and of both species (*p* < 0.05). All data analyses were performed using STATISTICA (version 6.0).

## Figures and Tables

**Table 1:** Morphometric characteristics of cysts of *Globodera pallida* and *G. rostochiensis* alone isolates from Algeria (*n* = 10 cysts). Measurements in μm and in the form: mean ± standard deviation (range).

Isolate Code	Body Length (L)	Body Width (W)	L/W Ratio	Neck Length	Number of Ridges	Distance Fenestra to Anus	Fenestra Diameter	Granek’s Ratio
**Algerian isolates**								
*Globodera pallida*								
9	508.0 ± 54.4 (422.7–588.2)	406.6 ± 57.8 (336.0–496.0)	1.24 ± 0.07 (1.15–1.38)	113.0 ± 33.9 (71.1–190.0)	12.6 ± 3.0 (8–18)	53.2 ± 7.8 (38.5–63.7)	19.7 ± 4.2 (14.1–26.8)	2.7 ± 0.4 (2.3–3.2)
10	502.5 ± 110.7 (346.3–727.0)	474.3 ± 86.6 (337.7–631.7)	1.05 ± 0.08 (0.91–1.17)	100.5 ± 53.2 (35.5–197.6)	14.0 ± 4.3 (8–19)	63.4 ± 20.0 (38.2–91.1)	23.2 ± 5.3 (17.1–30.1)	2.7 ± 0.6 (1.4–3.4)
11	570.0 ± 45.7 (499.2–632.4)	547.5 ± 61.6 (454.0–633.1)	1.04 ± 0.05 (0.97–1.15)	108.9 ± 41.2 (60.2–169.0)	12.3 ± 3.0 (8–18)	57.1 ± 15.0 (38.4–89.4)	21.0 ± 4.1 (16.0–27.9)	2.7 ± 0.4 (1.9–3.3)
12	539.7 ± 61.8 (468.1–671.5)	516.8 ± 64.4 (443.8–654.4)	1.04 ± 0.08 (0.93–1.22)	86.1 ± 29.1 (45.3–150.3)	13.6 ± 3.3 (9–18)	59.7 ± 14.0 (39.3–85.1)	23.5 ± 6.0 (16.4–33.4)	2.5 ± 0.3 (2.0–3.2)
13	543.8 ± 67.6 (477.5–627.7)	542.0 ± 74.1 (442.2–624.7)	1.00 ± 0.04 (0.94–1.07)	77.6 ± 25.9 (48.8–130.6)	14.0 ± 3.1 (8–18)	63.2 ± 13.6 (47.3–85.7)	23.2 ± 4.6 (18.0–30.5)	2.7 ± 0.2 (2.4–3.2)
14	570.8 ± 77.6 (375.4–638.7)	522.5 ± 80.0 (356.7–613.0)	1.09 ± 0.07 (1.00–1.26)	90.8 ± 46.7 (40.0–182.5)	13.0 ± 3.6 (9–19)	55.2 ± 12.0 (40.1–77.3)	23.0 ± 5.2 (18.0–34.8)	2.4 ± 0.3 (1.9–3.1)
15	550.8 ± 79.3 (473.6–704.5)	497.9 ± 65.6 (399.3–604.7)	1.10 ± 0.06 (0.99–1.21)	76.1 ± 23.1 (51.8–122.8)	12.2 ± 1.9 (10–16)	52.6 ± 7.4 (40.4–62.1)	21.3 ± 3.4 (15.7–25.9)	2.4 ± 0.3 (1.9–3.0)
16	489.5 ± 56.6 (405.2–566.9)	469.7 ± 66.8 (365.4–548.3)	1.04 ± 0.05 (0.91–1.10)	78.5 ± 30.9 (42.2–130.5)	13.6 ± 2.1 (11–17)	56.5 ± 9.1 (42.7–68.7)	23.3 ± 6.3 (14.4–32.9)	2.5 ± 0.4 (1.8–3.2)
17	550.4 ± 72.7 (436.2–638.8)	522.4 ± 65.3 (395.0–588.7)	1.05 ± 0.06 (0.92–1.14)	83.1 ± 21.1 (63.0–133.6)	12.0 ± 3.2 (8–18)	56.1 ± 8.1 (44.5–69.4)	23.4 ± 5.0 (16.8–30.3)	2.4 ± 0.5 (2.0–3.5)
18	484.9 ± 80.7 (364.2–609.5)	467.2 ± 82.3 (336.5–580.7)	1.03 ± 0.08 (0.92–1.19)	71.1 ± 26.6 (32.9–98.1)	12.5 ± 2.5 (9–17)	58.9 ± 10.1 (45.3–73.0)	22.1 ± 4.8 (15.0–31.6)	2.7 ± 0.5 (1.9–3.4)
19	479.0 ± 47.6 (414.6–560.1)	472.6 ± 58.1 (365.3–560.0)	1.01 ± 0.05 (0.95–1.13)	67.6 ± 34.0 (27.2–114.6)	11.9 ± 3.1 (8–16)	55.8 ± 15.2 (36.4–84.4)	23.2 ± 6.9 (13.9–31.2)	2.4 ± 0.5 (1.9–3.5)
20	496.9 ± 81.8 (348.6–599.2)	450.0 ± 88.7 (311.4–601.2)	1.10 ± 0.08 (0.99–1.29)	84.9 ± 33.3 (44.8–160.8)	14.0 ± 2.5 (11–18)	61.6 ± 9.6 (44.0–76.9)	20.7 ± 3.4 (14.6–27.4)	2.9 ± 0.4 (2.4–3.5)
21	526.5 ± 109.3 (327.0–676.9)	499.3 ± 101.6 (314.1–621.9)	1.05 ± 0.04 (1.00–1.14)	75.7 ± 19.5 (44.3–99.3)	11.7 ± 2.5 (8–15)	59.0 ± 11.7 (38.1–75.8)	22.7 ± 3.5 (16.0–28.2)	2.6 ± 0.4 (1.7–3.1)
22	476.2 ± 69.3 (380.0–558.8)	448.9 ± 64.6 (389.4–519.5)	1.06 ± 0.08 (0.92–1.21)	104.7 ± 45.0 (49.2–178.8)	13.4 ± 2.7 (9–18)	63.5 ± 11.2 (48.9–87.9)	23.4 ± 4.5 (16.8–30.4)	2.7 ± 0.3 (2.2–3.3)
26	560.1 ± 58.2 (478.6–668.8)	523.0 ± 53.4 (452.2–639.8)	1.06 ± 0.05 (1.00–1.14)	118.9 ± 25.8 (71.3–150.2)	12.0 ± 3.1 (9–18)	54.2 ± 15.0 (37.2–78.8)	23.9 ± 4.6 (17.1–31.0)	2.2 ± 0.3 (1.8–3.0)
29	457.0 ± 54.0 (392.9–547.9)	418.4 ± 63.2 (316.4–509.3)	1.07 ± 0.08 (0.96–1.22)	106.3 ± 36.4 (71.4–195.2)	12.1 ± 1.9 (9–15)	52.4 ± 10.0 (39.3–70.6)	21.3 ± 4.3 (15.0–28.4)	2.5 ± 0.6 (1.6–3.4)
*Globodera rostochiensis*								
3	596.8 ± 65.2 (520.9–703.2)	560.7 ± 64.2 (481.5–568.2)	1.06 ± 0.04 (0.96–1.14)	107.2 ± 39.0 (55.6–159.9)	18.0 ± 2.8 (16–24)	64.1 ± 12.1 (50.6–90.1)	19.3 ± 3.8 (13.2–24.4)	3.4 ± 1.0 (2.2–5.9)
7	505.0 ± 85.5 (341.7–587.1)	458.5 ± 80.8 (312.4–568.1)	1.09 ± 0.06 (1.03–1.21)	135.5 ± 39.3 (83.5–177.8)	19.0 ± 4.4 (13–25)	71.9 ± 17.3 (45.3–93.8)	17.5 ± 3.9 (13.6–26.7)	4.1 ± 1.0 (2.5–5.5)
23	492.0 ± 55.6 (399.1–566.8)	462.5 ± 79.9 (318.8–588.0)	1.07 ± 0.08 (0.96–1.25)	89.7 ± 26.9 (57.8–127.0)	18.5 ± 2.6 (15–23)	62.8 ± 11.1 (44.8–87.7)	18.2 ± 4.1 (14.0–25.0)	3.6 ± 1.0 (2.1–5.2)
24	466.0 ± 119.4 (302.4–617.1)	457.4 ± 115.8 (301.5–607.7)	1.01 ± 0.06 (0.91–1.11)	89.7 ± 26.9 (45.0–150.6)	20.0 ± 4.4 (13–26)	75.1 ± 20.4 (51.6–18.4)	19.7 ± 4.7 (14.0–28.5)	4.0 ± 1.4 (3.0–6.7)
25	463.8 ± 59.5 (346.1–570.8)	430.8 ± 62.8 (367.9–594.1)	1.01 ± 0.04 (0.96–1.07)	76.9 ± 26.0 (27.2–105.1)	18.3 ± 2.8 (12–21)	60.3 ± 5.9 (46.9–68.0)	17.1 ± 3.3 (13.6–23.1)	3.6 ± 0.8 (2.4–4.7)
27	528.3 ± 80.2 (366.7–654.1)	514.7 ± 88.7 (344.0–635.5)	1.02 ± 0.05 (0.93–1.12)	96.2 ± 40.0 (43.6–150.4)	22.0 ± 3.3 (17–28)	92.6 ± 27.2 (60.1–53.6)	22.0 ± 4.0 (17.3–27.9)	4.2 ± 1.2 (2.9–6.2)
28	563.8 ± 57.3 (493.6–660.9)	561.7 ± 43.3 (474.5–614.5)	1.00 ± 0.08 (0.92–1.15)	76.5 ± 23.0 (46.1–114.4)	22.2 ± 4.2 (16–28)	99.9 ± 28.1 (65.0–45.3)	22.3 ± 3.9 (15.9–27.9)	4.5 ± 1.1 (3.3–6.7)
30	537.2 ± 88.9 (373.5–669.4)	497.1 ± 77.1 (357.9–599.6)	1.07 ± 0.06 (0.95–1.17)	60.8 ± 19.2 (40.6–97.3)	21.7 ± 3.7 (16–27)	90.1 ± 23.7 (57.9–40.4)	22.1 ± 3.5 (16.7–27.6)	4.2 ± 1.5 (2.4–6.7)
32	527.9 ± 49.0 (458.0–621.3)	509.6 ± 50.7 (441.6–599.0)	1.03 ± 0.02 (1.00–1.08)	78.8 ± 23.0 (47.4–127.1)	17.7 ± 4.2 (12–23)	59.6 ± 8.7 (48.5–75.7)	19.8 ± 3.5 (14.3–24.8)	3.1 ± 0.9 (2.4–5.2)
34	496.4 ± 72.4 (394.0–634.7)	469.8 ± 72.2 (368.8–632.7)	1.05 ± 0.05 (1.00–1.16)	84.4 ± 19.7 (65.7–122.4)	17.0 ± 4.0 (12–24)	60.6 ± 13.8 (40.9–80.7)	17.0 ± 2.7 (13.6–22.4)	3.5 ± 0.5 (2.9–4.6)
**Reference measurements**								
*Globodera pallida*								
[40]	*	*	*	*	8–20 (<14)	22–67 (<50)	18–21 (>19)	1.2–3.5 (<3)
[47]	*	*	*	*	8–20 (<14)	*	*	1.2–3.5 (<3)
Composite	*	*	*	*	8–20	22–67	18–21	1.2–3.5
*Globodera rostochiensis*								
[40]	*	*	*	*	12–31 (>14)	37–77 (>55)	8–20 (<19)	1.3–9.5 (>3)
[47]	*	*	*	*	16–31 (>14)	*	*	1.3–9.5 (>3)
Composite	*	*	*	*	12–31	37–77	8–20	1.3–9.5

* no data.

**Table 2:** Morphometric characteristics of second-stage juveniles of *Globodera pallida* and *G. rostochiensis* alone isolates from Algeria (*n* = 10 J2s). Measurements in μm and in the form: mean ± SD (range).

Isolate Code	Stylet Length	Stylet Knobs Shape ^a^	Body Length	Body Width in the Middle	Body Width at the Anus	Tail Length	Hyaline Part of Tail Length	a	c	c′
**Algerian isolates**										
*Globodera pallida*										
9	24.3 ± 1.2 (23.0–27.2)	1	435.3 ± 25.4 (401.0–489.6)	19.7 ± 1.0 (18.4–21.5)	11.9 ± 0.4 (11.4–12.5)	47.0 ± 2.3 (43.4–49.7)	26.2 ± 1.7 (22.8–29.4)	22.0 ± 1.2 (19.1–23.3)	9.1 ± 0.6 (8.1–9.9)	3.9 ± 0.2 (3.4–4.2)
10	23.8 ± 1.1 (22.0–25.3)	1	422.2 ± 17.7 (388.9–444.9)	19.0 ± 1.1 (17.1–20.6)	11.5 ± 0.5 (10.8–12.1)	47.1 ± 2.1 (42.2–49.2)	24.3 ± 1.9 (21.1–27.5)	22.2 ± 1.1 (20.1–23.8)	8.9 ± 0.3 (8.5–9.5)	4.0 ± 0.1 (3.8–4.4)
11	24.4 ± 0.8 (23.1–25.6)	1	434.0 ± 18.7 (405.8–463.9)	19.5 ± 1.1 (17.3–21.6)	11.9 ± 0.5 (10.6–12.7)	49.7 ± 4.1 (44.0–54.6)	27.9 ± 2.7 (22.5–31.1)	22.2 ± 1.1 (20.8–23.7)	8.7 ± 0.7 (8.0–0.2)	4.1 ± 0.4 (3.4–4.6)
12	24.3 ± 1.0 (23.0–26.0)	1	457.7 ± 18.0 (413.8–487.1)	19.7 ± 0.8 (18.5–21.1)	12.0 ± 0.4 (11.1–12.4)	47.5 ± 2.7 (43.8–52.4)	26.4 ± 2.0 (23.0–29.9)	23.2 ± 1.3 (21.4–25.4)	9.8 ± 0.6 (8.3–0.9)	3.9 ± 0.2 (3.6–4.3)
13	23.4 ± 0.8 (22.0–24.6)	1	432.9 ± 36.5 (382.0–499.2)	19.0 ± 1.2 (17.2–21.0)	11.4 ± 0.5 (10.6–12.3)	45.0 ± 5.0 (38.3–52.8)	25.4 ± 3.3 (21.3–29.6)	22.6 ± 1.0 (21.0–23.8)	9.6 ± 1.0 (8.4–0.8)	3.9 ± 0.4 (3.3–4.7)
14	24.3 ± 1.1 (22.3–25.9)	1	457.1 ± 34.8 (417.1–514.3)	20.1 ± 1.3 (17.4–21.6)	12.3 ± 0.5 (11.2–12.8)	48.0 ± 5.0 (38.2–54.8)	27.8 ± 2.2 (23.2–31.2)	22.6 ± 1.5 (20.4–25.7)	9.5 ± 0.9 (8.7–1.7)	3.8 ± 0.3 (3.4–4.2)
15	24.1 ± 1.2 (22.1–26.0)	1	453.1 ± 25.4 (414.0–490.2)	20.0 ± 1.2 (18.2–21.5)	12.1 ± 0.4 (11.5–12.8)	50.2 ± 2.4 (46.5–54.8)	29.1 ± 2.1 (26.1–31.2)	22.5 ± 0.5 (21.7–23.5)	9.0 ± 0.5 (8.4–9.8)	4.1 ± 0.2 (3.8–4.3)
16	24.1 ± 1.4 (21.7–26.1)	1	461.9 ± 33.5 (398.2–509.4)	20.0 ± 1.2 (18.2–21.5)	12.2 ± 0.2 (11.8–12.6)	50.6 ± 4.3 (44.1–57.5)	29.0 ± 2.0 (25.4–31.2)	23.0 ± 1.3 (21.0–24.6)	9.1 ± 0.7 (8.2–0.6)	4.1 ± 0.3 (3.6–4.7)
17	24.5 ± 1.3 (23.1–27.6)	1	458.9 ± 23.1 (418.0–492.7)	19.9 ± 1.1 (18.1–21.6)	12.1 ± 0.3 (11.6–12.5)	50.8 ± 4.5 (41.7–57.1)	26.7 ± 2.5 (24.3–31.7)	23.0 ± 1.3 (21.4–25.1)	9.0 ± 0.7 (7.6–0.6)	4.2 ± 0.3 (3.4–4.6)
18	24.4 ± 0.6 (23.3–25.4)	1	435.3 ± 38.9 (391.1–504.1)	19.5 ± 1.6 (17.1–21.4)	11.8 ± 0.5 (10.7–12.5)	48.7 ± 2.7 (45.7–53.7)	27.4 ± 1.8 (25.0–30.3)	22.3 ± 1.1 (19.8–23.8)	8.9 ± 0.8 (7.8–0.3)	4.1 ± 0.2 (3.6–4.6)
19	24.4 ± 0.8 (22.6–25.2)	1	440.5 ± 13.1 (421.0–461.0)	19.4 ± 0.9 (18.0–20.9)	11.8 ± 0.5 (11.1–12.7)	47.4 ± 3.4 (43.9–54.8)	27.6 ± 2.3 (24.6–31.1)	22.7 ± 1.0 (20.4–24.2)	9.3 ± 0.5 (7.9–9.7)	3.9 ± 0.3 (3.5–4.7)
20	23.7 ± 0.8 (21.7–24.4)	1	444.5 ± 20.5 (400.6–471.0)	19.5 ± 1.0 (17.9–21.4)	11.9 ± 0.3 (11.2–12.4)	45.6 ± 1.7 (43.4–48.2)	23.9 ± 2.6 (21.1–28.7)	22.7 ± 0.7 (21.7–23.9)	9.7 ± 0.5 (9.0–0.7)	3.8 ± 0.1 (3.5–4.2)
21	24.3 ± 1.0 (23.3–26.6)	1	451.3 ± 25.5 (423.2–496.1)	20.1 ± 1.0 (18.2–21.5)	12.1 ± 0.3 (11.6–12.6)	50.7 ± 3.1 (45.0–52.7)	27.8 ± 2.5 (24.1–31.8)	22.4 ± 1.1 (20.6–24.4)	8.9 ± 0.5 (8.2–9.9)	4.1 ± 0.2 (3.5–4.5)
22	23.9 ± 0.7 (22.4–25.1)	1	448.5 ± 35.7 (401.7–514.2)	20.0 ± 1.1 (18.2–21.4)	11.9 ± 0.4 (11.1–12.5)	45.5 ± 4.9 (35.8–51.8)	25.0 ± 2.9 (21.2–30.4)	22.3 ± 1.1 (19.5–24.0)	9.9 ± 0.9 (8.4–1.9)	3.8 ± 0.4 (3.0–4.3)
26	23.7 ± 1.1 (22.0–25.4)	1	450.9 ± 37.4 (392.3–497.6)	19.7 ± 1.1 (17.5–21.2)	11.9 ± 0.3 (11.2–12.3)	45.5 ± 3.1 (40.2–50.0)	26.5 ± 2.6 (23.1–30.5)	22.8 ± 1.2 (21.1–24.7)	9.9 ± 1.0 (8.1–1.3)	3.8 ± 0.2 (3.5–4.1)
29	24.0 ± 0.8 (22.3–24.9)	1	425.0 ± 28.3 (394.0–471.8)	19.1 ± 1.2 (17.7–21.6)	11.4 ± 0.4 (11.0–12.4)	45.4 ± 1.8 (42.0–47.7)	25.2 ± 2.5 (20.4–29.0)	22.1 ± 0.7 (21.1–23.5)	9.3 ± 0.7 (8.6–0.8)	3.9 ± 0.2 (3.4–4.3)
*Globodera rostochiensis*										
3	22.0 ± 0.4 (21.1–22.7)	2	445.3 ± 27.5 (397.1–492.4)	19.4 ± 1.4 (17.2–21.6)	12.0 ± 0.5 (10.8–12.6)	48.1 ± 4.5 (40.6–54.0)	25.8 ± 2.6 (23.1–30.9)	22.9 ± 1.0 (20.3–24.2)	9.2 ± 0.5 (8.5–0.2)	4.0 ± 0.2 (3.5–4.3)
7	21.8 ± 0.7 (20.9–22.9)	2	439.8 ± 31.5 (393.8–488.6)	19.6 ± 0.9 (18.4–21.1)	11.8 ± 0.3 (11.4–12.4)	47.1 ± 4.5 (37.5–52.3)	26.5 ± 2.6 (22.0–30.4)	22.4 ± 1.3 (19.3–24.5)	9.3 ± 1.0 (8.2–1.1)	3.9 ± 0.4 (3.2–4.5)
23	21.9 ± 0.7 (20.8–23.1)	2	442.2 ± 34.3 (391.3–485.0	18.9 ± 1.0 (17.5–20.5)	11.7 ± 0.5 (11.0–12.4)	44.7 ± 4.1 (37.5–49.4)	23.9 ± 2.0 (21.2–27.0)	23.1 ± 1.4 (20.0–25.0)	9.9 ± 0.3 (9.2–0.4)	3.8 ± 0.2 (3.4–4.3)
24	21.9 ± 0.7 (21.1–23.3)	2	434.9 ± 29.4 (382.7–477.7)	18.7 ± 1.0 (17.4–20.8)	11.5 ± 0.5 (10.6–12.1)	44.7 ± 6.0 (35.8–53.6)	24.3 ± 3.7 (20.4–31.0)	23.2 ± 1.2 (21.9–24.3)	9.8 ± 0.8 (8.5–0.7)	3.8 ± 0.5 (3.3–4.6)
25	22.2 ± 0.6 (21.1–23.1)	2	443.5 ± 43.3 (379.1–508.6)	19.5 ± 1.5 (16.9–21.5)	11.7 ± 0.6 (10.4–12.5)	46.6 ± 2.2 (43.5–50.5)	25.8 ± 2.4 (22.8–30.1)	22.7 ± 1.0 (20.8–24.6)	9.5 ± 1.0 (8.0–1.4)	3.9 ± 0.2 (3.6–4.4)
27	23.0 ± 0.4 (22.3–23.5)	2	442.9 ± 27.2 (400.5–487.3)	19.4 ± 1.0 (18.1–20.8)	11.6 ± 0.3 (11.1–12.0)	45.3 ± 3.8 (40.7–51.2)	26.4 ± 2.1 (24.2–30.7)	22.8 ± 0.6 (21.8–23.5)	9.7 ± 0.5 (8.5–0.6)	3.8 ± 0.2 (3.5–4.2)
28	23.0 ± 0.5 (22.0–23.5)	2	464.9 ± 34.3 (393.5–500.0)	20.2 ± 1.2 (18.5–21.6)	12.4 ± 0.5 (11.1–12.9)	50.6 ± 3.2 (44.8–53.7)	29.4 ± 2.1 (25.1–32.1)	22.9 ± 0.7 (21.1–23.8)	9.1 ± 0.5 (8.4–0.0)	4.0 ± 0.1 (3.7–4.3)
30	23.0 ± 0.5 (21.7–23.5)	2	456.0 ± 20.6 (419.4–482.6)	20.0 ± 1.0 (18.1–21.2)	12.3 ± 0.4 (11.2–12.8)	50.4 ± 3.4 (44.4–55.7)	27.6 ± 2.7 (23.9–31.4)	22.8 ± 0.73 (21.7–24.4)	9.0 ± 0.7 (8.0–0.1)	4.1 ± 0.3 (3.4–4.6)
32	21.3 ± 0.8 (20.1–22.9)	2	440.6 ± 34.0 (390.7–503.1)	19.0 ± 0.6 (17.6–20.7)	11.8 ± 0.7 (10.4–12.5)	43.2 ± 4.0 (37.2–51.0)	23.6 ± 2.4 (20.8–28.8)	22.8 ± 1.1 (20.8–24.2)	10.2 ± 0.5 (8.9–0.8)	3.6 ± 0.4 (3.1–4.8)
34	21.9 ± 0.7 (20.0–22.7)	2	413.4 ± 7.8 (404.7–424.8	18.6 ± 0.5 (17.4–19.3)	11.3 ± 0.4 (10.8–12.0)	42.1 ± 4.4 (37.3–50.9)	23.4 ± 2.3 (20.9–28.5)	22.2 ± 0.6 (21.3–23.2)	9.9 ± 1.0 (8.3–1.2)	3.6 ± 0.3 (3.2–4.2)
**Reference measurements**										
*Globodera pallida*										
[40]	21–26 (>23)	1	*	*	*	*	*	*	*	*
[47]	23.8 (22–24)	1	484 (440–525)	*	*	*	*	*	*	*
Composite	21–26	1	440–525	*	*	*	*	*	*	*
*Globodera rostochiensis*										
[40]	21–23 (22)	2	*	*	*	*	*	*	*	*
[47]	21.8 (19–23)	2	468 (425–505)	*	*	*	*	*	*	*
Composite	19–23	2	425–505	*	*	*	*	*	*	*

^a^: 1, pointed knobs correspond to *Globodera pallida*; 2, rounded knobs correspond to *G. rostochiensis.* *: no data. a (body length/body width in the middle), c (body length/tail length) and c′ (tail length/body width at anus level).

**Table 3:** Morphometric characteristics of cysts of *Globodera pallida and G. rostochiensis* mixed isolates from Algeria (*n* = 10 cysts). Measurements in μm and in the form: mean ± SD (range).

Isolate Code		Body Length (L)	Body Width (W)	L/W Ratio	Neck Length	Number of Ridges	Distance Fenestra to Anus	Fenestra Diameter	Granek’s Ratio	Morphological Identification
1	*n* = 4	546.6 ± 67.9 (452.9–613.3)	463.1 ± 36.7 (408.5–487.8)	1.12 ± 0.04 (1.07–1.18)	76.4 ± 19.7 (48.6–95.2)	11.5 ± 2.3 (9–14)	53.2 ± 8.4 (41.6–60.6)	20.5 ± 4.3 (16.6–24.4)	2.6 ± 0.6 (2.1–3.5)	*G. pallida*
	*n* = 6	561.5 ± 48.3 (489.4–614.2)	543.2 ± 70.6 (421.0–611.3)	1.06 ± 0.08 (0.97–1.16)	70.8 ± 23.8 (48.8–13.1)	16.3 ± 3.7 (12–23)	77.1 ± 18.7 (59.8–12.6)	20.5 ± 2.5 (18.2–24.4)	3.8 ± 1.3 (2.4 ± 6.1)	*G. rostochiensis*
2	*n* = 6	555.3 ± 78.8 (425.2–638.2)	509.9 ± 79.4 (384.3–575.6)	1.09 ± 0.08 (1.01–1.25)	93.6 ± 40.8 (37.1–27.0)	11.0 ± 1.2 (10–13)	50.3 ± 5.9 (43.5–58.6)	16.7 ± 3.0 (13.5–20.3)	3.0 ± 0.4 (2.1–3.4)	*G. pallida*
	*n* = 4	530.7 ± 98.9 (421.8–649.1)	507.4 ± 80.5 (395.5–587.3)	1.04 ± 0.07 (0.93–1.10)	100.7 ± 62.7 (43.5–82.8)	15.0 ± 1.4 (14–17)	56.0 ± 8.9 (49.0–68.6)	15.3 ± 1.2 (15.0–16.7)	3.6 ± 0.3 (3.2 ± 4.1)	*G. rostochiensis*
4	*n* = 3	514.3 ± 24.5 (491.4–540.3)	477.5 ± 33.2 (446.0–512.2)	1.07 ± 0.02 (1.05–1.10)	101.2 ± 22.2 (75.6–15.7)	13.3 ± 4.1 (10–18)	58.9 ± 17.6 (43.0–77.9)	23.0 ± 2.2 (20.5–24.9)	2.5 ± 0.5 (2.0–3.1)	*G. pallida*
	*n* = 7	555.2 ± 80.4 (431.9–650.3)	493.8 ± 81.0 (391.2–587.7)	1.12 ± 0.07 (1.05–1.26)	102.8 ± 34.7 (64.0–54.7)	17.7 ± 4.3 (12–21)	70.8 ± 12.3 (63.2–90.2)	15.8 ± 2.5 (13.4–19.6)	4.5 ± 0.9 (3.3–6.4)	*G. rostochiensis*
5	*n* = 3	518.0 ± 43.2 (468.6–549.1)	472.5 ± 38.6 (449.4–517.1)	1.09 ± 0.07 (1.04–1.18)	94.3 ± 29.8 (73.6–28.6)	14.3 ± 3.0 (11–17)	59.7 ± 12.3 (45.7–68.9)	20.2 ± 2.0 (18.7–22.5)	3.0 ± 0.8 (2.0–3.5)	*G. pallida*
	*n* = 7	528.6 ± 44.6 (465.4–587.5)	524.9 ± 66.2 (431.8–618.0)	1.00 ± 0.04 (0.95–1.07)	87.6 ± 49.4 (40.9–90.1)	15.8 ± 3.9 (12–22)	78.8 ± 13.2 (65.6–00.5)	15.9 ± 2.1 (13.6–18.3)	4.9 ± 0.8 (3.6–6.1)	*G. rostochiensis*
6	*n* = 6	578.5 ± 47.2 (518.0–638.1)	482.5 ± 111.6 (309.4–609.0)	1.08 ± 0.02 (1.04–1.11)	89.6 ± 46.9 (42.0–59.3)	10.3 ± 1.5 (8–12)	51.5 ± 1.3 (49.4–53.3)	17.1 ± 3.5 (13.9–23.2)	3.0 ± 0.5 (2.1–3.5)	*G. pallida*
	*n* = 4	516.1 ± 62.6 (458.9–603.9)	456.4 ± 85.8 (333.3–523.3)	1.06 ± 0.05 (1.01–1.15)	76.0 ± 17.8 (51.9–94.9)	15.7 ± 2.2 (13–18)	77.5 ± 11.7 (62.8–90.3)	17.3 ± 4.3 (13.1–22.0)	4.6 ± 0.9 (3.7–5.8)	*G. rostochiensis*
8	*n* = 4	636.2 ± 115.4 (468.4–732.2)	550.6 ± 82.5 (460.6–622.8)	1,16 ± 0,01 (1,14–1,17)	131.5 ± 30.6 (91.8–66.6)	10.2 ± 1.2 (9–12)	53.1 ± 2.3 (51.0–56.4)	21.5 ± 2.7 (19.2–25.3)	2.4 ± 0.3 (2.1–2.9)	*G. pallida*
	*n* = 6	556.7 ± 102.9 (418.0–75.10)	526.5 ± 87.0 (396.8–621.4)	1.05 ± 0.03 (0.99–1.09)	95.4 ± 61.1 (41.1–91.7)	19.1 ± 3.3 (15–24)	82.2 ± 14.7 (63.8–02.3)	19.2 ± 3.1 (14.4–22.2)	4.2 ± 0.3 (3.5–4.5)	*G. rostochiensis*
31	*n* = 6	590.9 ± 47.0 (536.5–653.3)	574.0 ± 55.2 (500.0–635.5)	1.02 ± 0.04 (0.99–1.08)	109.9 ± 35.6 (71.6–53.2)	13.0 ± 4.3 (8–20)	59.9 ± 16.4 (40.2–79.0)	21.8 ± 3.4 (17.1–25.6)	2.7 ± 0.5 (2.1–3.2)	*G. pallida*
	*n* = 4	611.3 ± 53.9 (548.8–674.5)	589.8 ± 44.0 540.9–640.3)	1.03 ± 0.05 (0.96–1.09)	95.0 ± 29.2 (55.3–23.5)	16.7 ± 4.5 (12–22)	74.9 ± 21.0 (48.1–96.2)	20.2 ± 2.9 (16.0–23.0)	4.1 ± 1.2 (3.0–5.9)	*G. rostochiensis*
33	*n* = 4	536.4 ± 47.6 (468.7–572.5)	495.5 ± 45.6 (444.6–551.2)	1.08 ± 0.08 (1.02–1.20)	90.8 ± 15.0 (71.0–07.5)	8.5 ± 0.5 (8–9)	40.5 ± 2.0 (38.2–42.3)	17.1 ± 2.2 (15.0–19.9)	2.3 ± 0.3 (2.1–2.7)	*G. pallida*
	*n* = 6	585.4 ± 74.1 (497.4–674.0)	543.8 ± 60.9 (453.1–614.3)	1.07 ± 0.08 (1.01–1.23)	105.8 ± 35.2 (65.5–65.1)	15.5 ± 4.4 (12–23)	67.4 ± 22.5 (42.0–03.2)	18.0 ± 3.8 (13.4–24.4)	3.7 ± 1.2 (2.4–5.7)	*G. rostochiensis*
35	*n* = 4	469.0 ± 20.5 (439.0–483.2)	433.8 ± 33.2 (400.4–479.1)	1.09 ± 0.03 (1.07–1.14)	83.1 ± 23.6 (55.0–12.4)	9.0 ± 1.4 (8–11)	46.1 ± 4.8 (40.6–51.8)	21.3 ± 2.4 (17.7–23.3)	2.1 ± 0.1 (2.0–2.2)	*G. pallida*
	*n* = 6	537.8 ± 43.3 (473.7–595.2)	483.7 ± 40.1 (446.1–546.6)	1.09 ± 0.05 (1.00–1.15)	92.2 ± 26.8 (58.5–29.0)	14.8 ± 5.1 (12–25)	69.4 ± 26.9 (49.9–22.0)	17.5 ± 3.6 (13.2–21.3)	3.9 ± 0.9 (3.0–5.7)	*G. rostochiensis*
36	*n* = 3	589.6 ± 34.3 (566.0–629.1)	576.8 ± 38.2 (546.4–619.7)	1.01 ± 0.01 (1.01–1.03)	82.8 ± 15.3 (68.3–98.9)	9.0 ± 1.0 (8–10)	42.5 ± 9.1 (32.1–49.2)	15.6 ± 0.4 (15.1–16.0)	2.7 ± 0.6 (2.0–3.1)	*G. pallida*
	*n* = 7	522.2 ± 82.0 (443.8–667.4)	459.6 ± 77.3 (366.1–583.7)	1.13 ± 0.07 (1.06–1.25)	110.9 ± 20.7 (82.8–37.7)	16.2 ± 4.4 (12–24)	79.0 ± 28.1 (52.5–30.6)	18.0 ± 2.2 (15.2–20.7)	4.3 ± 1.4 (2.8–6.5)	*G. rostochiensis*

**Table 4:** Morphometric characteristics of second-stage juveniles of *Globodera pallida* and *G. rostochiensis* mixed isolates from Algeria (*n* = 10 J2s). Measurements in μm and in the form: mean ± SD (range).

Isolates Code		Stylet Length	Stylet Knobs Shape ^a^	Body Length	Body Width in the Middle	Body Width at the Anus	Tail Length	Hyaline Part of Tail Length	a	c	c′	Morphological Identification
1	*n* = 4	24.6 ± 1.0 (23.2–25.8)	1	427.3 ± 40.2 (391.8–473.9)	19.6 ± 0.9 (18.3–20.5)	12.1 ± 0.3 (11.9–12.6)	46.2 ± 2.9 (42.5–48.8)	23.3 ± 1.0 (22.2–24.4)	21.6 ± 1.3 (19.9–23.0)	9.2 ± 0.4 (8.6–9.7)	3.8 ± 0.2 (3.5–4.0)	*G. pallida*
	*n* = 6	21.9 ± 0.8 (20.3–22.9)	2	452.7 ± 28.4 (420.7–494.2)	19.3 ± 1.2 (18.2–21.0)	11.6 ± 0.7 (11.0–12.6)	50.3 ± 2.8 (46.6–54.5)	26.3 ± 2.2 (23.2–29.3)	23.3 ± 0.4 (22.8–24.0)	9.0 ± 0.8 (8.3–0.5)	4.3 ± 0.3 (3.7–4.7)	*G. rostochiensis*
2	*n* = 7	23.1 ± 0.6 (22.2–23.8)	1	419.1 ± 14.3 (390.8–433.1)	19.4 ± 0.7 (18.7–20.7)	11.9 ± 0.2 (11.5–12.3)	43.4 ± 4.1 (37.7–46.9)	23.5 ± 2.3 (20.8–27.9)	21.5 ± 1.3 (19.4–22.7)	9.7 ± 0.9 (8.8–0.7)	3.6 ± 0.3 (3.2–3.9)	*G. pallida*
	*n* = 3	22.3–1.5 (20.5–23.4)	2	422.7 ± 31.1 (401.6–458.5)	19.3 ± 1.2 (18.0–20.3)	12.0 ± 0.3 (11.7–12.4)	43.7 ± 4.6 (39.0–48.2)	25.2 ± 1.2 (23.9–26.4)	21.8 ± 1.3 (20.2–22.6)	9.7 ± 1.2 (8.3–0.4)	3.6 ± 0.2 (3.3–3.8)	*G. rostochiensis*
4	*n* = 4	24.1 ± 0.6 (23.4–24.7)	1	403.0 ± 8.7 (397.0–415.9)	19.2 ± 0.6 (18.7–20.1)	11.5 ± 0.2 (11.3–11.9)	42.3 ± 2.7 (39.4–45.9)	22.8 ± 2.8 (20.1–26.0)	20.9 ± 1.0 (19.7–22.2)	9.5 ± 0.7 (8.6–0.5)	3.6 ± 0.2 (3.3–3.9)	*G. pallida*
	*n* = 6	22.2 ± 0.7 (20.8–22.9)	2	404.2 ± 13.2 (381.1–420.3)	18.5 ± 1.0 (16.9–19.6)	11.5 ± 0.8 (10.3–12.4)	41.9 ± 2.4 (39.1–44.9)	23.9 ± 2.3 (20.1–27.1)	21.8 ± 1.0 (20.2–22.8)	9.6 ± 0.6 (8.8–0.4)	3.6 ± 0.3 (3.1–3.9)	*G. rostochiensis*
5	*n* = 4	22.9 ± 0.5 (22.2–23.5)	1	437.5 ± 4.7 (431.3–441.6)	20.3 ± 0.3 (19.8–20.7)	12.0 ± 0.5 11.4–12.7)	49.2 ± 4.4 (45.1–54.1)	26.0 ± 2.1 (22.8–27.6)	21.5 ± 0.3 (21.2–21.9)	8.9 ± 0.8 (8.1–9.6)	4.0 ± 0.4 (3.5–4.5)	*G. pallida*
	*n* = 6	21.5 ± 0.5 (20.7–22.2)	2	427.3 ± 25.8 (389.5–459.3)	19.4 ± 0.6 (18.3–19.9)	11.5 ± 0.3 (11.1–12.0)	42.3 ± 3.1 (38.7–47.0)	22.8 ± 3.1 (20.6–27.3)	21.9 ± 1.1 (20.3–23.7)	10.1 ± 0.7 (9.0–0.9)	3.6 ± 0.2 (3.3–4.0)	*G. rostochiensis*
6	*n* = 7	24.6 ± 0.7 (23.7–26.0)	1	420.4 ± 11.8 (403.5–440.6)	19.1 ± 1.2 (17.7–20.7)	11.5 ± 0.3 (11.1–11.9)	46.3 ± 1.8 (43.9–49.2)	25.7 ± 1.9 (23.4–29.2)	21.9 ± 1.1 (20.1–23.4)	9.0 ± 0.5 (8.1–0.0)	3.9 ± 0.2 (3.6–4.3)	*G. pallida*
	*n* = 3	22.4 ± 1.4 (20.8–23.3)	2	401.9 ± 14.9 (385.5–414.6)	18.3 ± 1.5 (16.9–19.9)	11.3 ± 0.7 (10.4–11.9)	46.4 ± 3.6 (43.0–50.2)	24.3 ± 1.7 (22.5–26.0)	21.9 ± 1.4 (20.3–22.7)	8.6 ± 0.6 (8.2–9.4)	4.1 ± 0.4 (3.6–4.4)	*G. rostochiensis*
8	*n* = 4	25.3 ± 1.1 (24.6–27.0)	1	434.2 ± 13.4 (414.4–444.7)	20.2 ± 0.5 (19.6–21.0)	12.3 ± 0.3 (11.9–12.7)	47.5 ± 0.4 (47.1–48.1)	23.8 ± 2.8 (21.3–27.6)	21.4 ± 0.9 (20.4–22.3)	9.1 ± 0.3 (8.6–9.4)	3.8 ± 0.1 (3.7–3.9)	*G. pallida*
	*n* = 6	22.7 ± 0.8 (21.2 ± 23.5)	2	416.0 ± 24.3 (393.1–452.6)	19.5 ± 0.9 (18.4–21.0)	11.9 ± 0.5 (11.2–12.7)	44.7 ± 3.9 (37.4–48.5)	24.0 ± 2.1 (20.9–26.6)	21.2 ± 0.9 (19.9–22.3)	9.3 ± 0.7 (8.7–0.7)	3.7 ± 0.3 (3.1–3.9)	*G. rostochiensis*
31	*n* = 6	22.6 ± 0.8 (22.0–24.2)	1	429.3 ± 13.8 (409.1–444.2)	19.5 ± 1.0 (18.0–21.2)	11.8 ± 0.6 (11.1–12.6)	49.3 ± 5.9 (40.9–55.3)	27.0 ± 2.7 (22.7–30.6)	21.9 ± 0.9 (20.6–23.3)	8.7 ± 1.0 (7.6–0.2)	4.1 ± 0.5 (3.4–4.8)	*G. pallida*
	*n* = 4	22.2 ± 0.5 (21.5–22.7)	2	398.6 ± 5.3 (394.4–406.4)	19.3 ± 1.1 (18.1–20.4)	11.7 ± 0.4 (11.1–12.2)	41.2 ± 2.7 (38.8–45.2)	22.7 ± 2.2 (20.6–25.2)	20.6 ± 1.0 (19.7–21.8)	9.6 ± 0.6 (8.7–0.2)	3.5 ± 0.2 (3.2–3.8)	*G. rostochiensis*
33	*n* = 4	23.1 ± 0.3 (22.9–23.6)	1	417.9 ± 29.5 (390.9–458.4)	18.9 ± 0.8 (18.3–20.0)	11.9 ± 0.5 (11.2–12.4)	41.6 ± 2.9 (39.1–45.6)	24.6 ± 2.1 (22.9–27.7)	22.0 ± 0.9 (21.1–22.9)	10.0 ± 0.4 (9.3–0.5)	3.4 ± 0.3 (3.2–3.7)	*G. pallida*
	*n* = 6	21.5 ± 0.8 (20.2–22.8)	2	408.3 ± 28.8 (379.2–456.2)	18.1 ± 1.1 (17.0–20.2)	11.3 ± 0.4 (10.7–12.0)	42.3 ± 2.0 (39.1–44.2)	23.7 ± 2.1 (20.6–25.9)	22.4 ± 0.3 (21.9–23.0)	9.6 ± 0.4 (9.1–0.3)	3.7 ± 0.1 (3.5–3.9)	*G. rostochiensis*
35	*n* = 3	24.9 ± 1.3 (23.9–26.5)	1	453.4 ± 19.8 (435.2–474.5)	19.1 ± 0.6 (18.3–19.5)	11.8 ± 0.7 (11.0–12.4)	50.6 ± 6.3 (43.3–54.7)	29.5 ± 2.6 (30.3–31.7)	23.7 ± 1.1 (22.4–24.5)	9.0 ± 0.8 (8.3–0.0)	4.2 ± 0.6 (3.6–4.8)	*G. pallida*
	*n* = 7	21.7 ± 0.8 (20.4–23.0)	2	429.9 ± 37.3 (372.2–466.7)	19.5 ± 1.5 (16.9–21.1)	11.9 ± 0.7 (10.6–12.6)	44.3 ± 5.4 (35.7–52.9)	22.7 ± 1.9 (20.1–25.5)	22.0 ± 1.0 (20.1–23.2)	9.7 ± 0.8 (8.5–1.1)	3.6 ± 0.4 (3.2–4.4)	*G. rostochiensis*
36	*n* = 4	23.5 ± 0.1 (23.4–23.7)	1	457.1 ± 11.8 (439.5–464.8)	19.5 ± 1.1 (18.5–20.8)	11.8 ± 0.4 (11.2–12.4)	46.7 ± 7.2 (38.3–55.3)	25.7 ± 1.4 (23.6–26.8)	23.3 ± 1.2 (22.1–24.9)	9.9 ± 1.3 (8.3–1.4)	3.9 ± 0.7 (3.2–4.9)	*G. pallida*
	*n* = 6	22.8 ± 0.7 (21.7–23.4)	2	446.8 ± 16.7 (423.7–463.2)	20.0 ± 1.2 (18.6–21.6)	12.3 ± 0.3 (11.9–12.8)	45.5 ± 6.2 (38.9–54.7)	23.4 ± 3.3 (20.1–26.8)	22.3 ± 1.3 (20.2–23.9)	9.9 ± 1.0 (8.4–1.0)	3.6 ± 0.4 (3.1–4.5)	*G. rostochiensis*

^a^: 1, pointed knobs correspond to *Globodera pallida*; 2, rounded knobs correspond to *G. rostochiensis.* a (body length/body width in the middle), c (body length/tail length) and c′ (tail length/body width at anus level).

**Table 5:** Morphometric features (in μm) of cysts and second-stage juveniles of *Globodera pallida* and *G. rostochiensis* samples from Algeria and comparison of mean values between isolates of each *Globodera* species and of both species.

Character	*Globodera pallida*		*Globodera rostochiensis*		*G. pallida* and *G. rostochiensis*
	Mean ± SD (Range)	*p* Value	Mean ± SD (Range)	*p* Value	*p* Value
**Cyst**	*n* = 203		*n* = 157		
Body length (L)	527.28 ± 77.78 (346.30–732.20)	0.0001 ***	527.59 ± 81.34 (302.40–703.20)	0.0007 ***	0.0001 ***
Body width (W)	492.31 ± 78.17 (311.40–654.40)	0.0001 ***	499.87 ± 79.62 (301.50–640.30)	0.0015 **	0.0001 ***
L/W ratio	1.07 ± 0.08 (0.91–1.38)	0.0001 ***	1.05 ± 0.06 (0.91–1.26)	0.0002 ***	0.0001 ***
Neck length	91.46 ± 35.43 (27.25–197.60)	0.0218 *	91.77 ± 35.65 (27.25–191.70)	0.0042 **	0.0008 ***
Number of ridges	12.43 ± 3.04 (8–19)	0.0185 *	18.33 ± 4.21 (12–28)	0.0004 ***	0.0001 ***
Distance fenestra to anus	56.45 ± 12.17 (32.19–91.10)	0.0956	73.83 ± 21.29 (40.94–153.60)	0.0001 ***	0.0001 ***
Fenestra diameter	21.82 ± 4.76 (13.54–34.85)	0.0692	18.90 ± 3.89 (13.13–28.54)	0.0003 ***	0.0001 ***
Granek’s ratio	2.62 ± 0.48 (1.40–3.59)	0.0457 *	4.00 ± 1.12 (2.12–6.76)	0.1413	0.0001 ***
**Second stage juvenile**	*n* = 207		*n* = 153		
Stylet length	24.07 ± 1.08 (21.72–27.63)	0.0020 **	22.20 ± 0.88 (20.02–23.58)	0.0001 ***	0.0001 ***
Stylet knobs shape ^a^	1		2		
Body length	440.68 ± 28.71 (382.05–514.30)	0.0004 ***	432.99 ± 46.52 (372.21–508.68)	0.0048 **	0.0001 ***
Body width in the middle	19.64 ± 1.12 (17.10–21.68)	0.5656	19.31 ± 1.16 (16.90–21.66)	0.0295 *	0.0426 *
Body width at the anus	11.91 ± 0.49 (10.65–12.81)	0.0003 ***	11.82 ± 0.59 (10.37–12.97)	0.0014 **	0.0001 ***
Tail length	47.46 ± 4.19 35.85–57.57	0.0001 ***	45.63 ± 4.78 (35.71–55.74)	0.0001 ***	0.0001 ***
Hyaline part of tail length	26.33 ± 2.83 (20.19–31.87)	0.0001 ***	25.07 ± 2.99 (20.14–32.11)	0.0001 ***	0.0001 ***
a	22.44 ± 1.18 (19.17–25.78)	0.0217 *	22.54 ± 1.13 (19.37–25.08)	0.0007 ***	0.0002 ***
c	9.32 ± 0.81 (7.62–11.93)	0.0047 **	9.60 ± 0.80 (8.04–11.43)	0.0305 *	0.0001 ***
c′	3.98 ± 0.35 3.00–4.91	0.0022 **	3.85 ± 0.37 (3.12–4.91)	0.0034 **	0.0001 ***

*p*: probability. *, significant difference (*p* < 0.05); **, highly significant difference (*p* < 0.01); ***, very highly significant difference (*p* < 0.001), and other differences are not significant (*p* > 0.05). ^a^: 1, pointed knobs correspond to *Globodera pallida*; 2, rounded knobs correspond to *G. rostochiensis.* a (body length/body width in the middle), c (body length/tail length) and c′ (tail length/body width at anus level).

**Table 6:** Cycle threshold (Ct) values for fluorescence of FAM and TET obtained in the DNA samples of Algeria potato cyst nematodes during the TaqMan real-time PCR test.

Isolate Code	Replicate	FAM		TET		*Globodera* spp. Identity
		Ct Value	Mean	Ct Value	Mean	
1	1	15.64	16.15	17.09	17.72	*G. pallida* *G. rostochiensis*
	2	16.66		18.35		
2	1	19.62	19.77	17.82	17.98	*G. pallida* *G. rostochiensis*
	2	19.93		18.13		
3	1	—	—	18.25	18.29	*G. rostochiensis*
	2	—		18.33		
4	1	17.94	18.39	19.86	20.49	*G. pallida* *G. rostochiensis*
	2	18.85		21.11		
5	1	24.35	24.52	22.33	22.43	*G. pallida* *G. rostochiensis*
	2	24.69		22.52		
6	1	14.52	14.81	16.28	16.55	*G. pallida* *G. rostochiensis*
	2	15.10		16.83		
7	1	—	—	18.98	19.12	*G. rostochiensis*
	2	—		19.26		
8	1	15.14	15.06	16.27	16.64	*G. pallida* *G. rostochiensis*
	2	14.98		17.00		
9	1	19.05	19.04	—	—	*G. pallida*
	2	19.03		—		
10	1	15.73	15.83	—	—	*G. pallida*
	2	15.92		—		
11	1	18.79	18.91	—	—	*G. pallida*
	2	19.03		—		
12	1	18.04	18.03	—	—	*G. pallida*
	2	18.03		—		
13	1	17.62	17.73	—	—	*G. pallida*
	2	17.83		—		
14	1	20.76	20.90	—	—	*G. pallida*
	2	21.05		—		
15	1	15.91	15.81	—	—	*G. pallida*
	2	15.72		—		
16	1	15.24	15.31	—	—	*G. pallida*
	2	15.37		—		
17	1	14,15	14.25	—	—	*G. pallida*
	2	14.35		—		
18	1	16.09	16.01	—	—	*G. pallida*
	2	15.94		—		
19	1	16.14	16.24	—	—	*G. pallida*
	2	16.35		—		
20	1	17.18	17.07	—	—	*G. pallida*
	2	16.96		—		
21	1	22.32	22.56	—	—	*G. pallida*
	2	22.80		—		
22	1	21.87	21.92	—	—	*G. pallida*
	2	21.98		—		
23	1	—	—	23.48	23.61	*G. rostochiensis*
	2	—		23.74		
24	1	—	—	18.30	18.49	*G. rostochiensis*
	2	—		18.68		
25	1	—	—	20.18	20.47	*G. rostochiensis*
	2	—		20.77		
26	1	20.91	20.90	—	—	*G. pallida*
	2	20.89		—		
27	1	—	—	22.43	22.75	*G. rostochiensis*
	2	—		23.07		
28	1	—	—	24.09	24.09	*G. rostochiensis*
	2	—		24.08		
29	1	28.50	29.19	—	—	*G. pallida*
	2	29.87		—		
30	1	—	—	18.32	18.62	*G. rostochiensis*
	2	—		18.91		
31	1	21.10	21.00	22.18	22.05	*G. pallida* *G. rostochiensis*
	2	20.89		21.92		
32	1	—	—	19.44	19.41	*G. rostochiensis*
	2	—		19.37		
33	1	18.80	18.92	19.88	20.02	*G. pallida* *G. rostochiensis*
	2	19.04		20.17		
34	1	—	—	18.56	18.87	*G. rostochiensis*
	2	—		19.18		
35	1	18.28	18.63	18.96	19.38	*G. pallida* *G. rostochiensis*
	2	18.97		19.80		
36	1	20.31	20.55	20.45	20.72	*G. pallida* *G. rostochiensis*
	2	20.80		20.98		

—: No fluorescence was observed.

**Table 7:** Characteristics of the sites prospected in potato producing areas of Algeria.

Position	Area	Locality	Latitude	Longitude	Altitude (m)	Variety	Isolate Code
Centre	Tipaza	Aïn Tagourait	36°36′13″ N	2°36′34″ E	19	Spunta	10
		Tipaza	36°35′31″ N	2°26′58″ E	12	Spunta	11
	Algiers	Zeralda - Field 1	36°43′5″ N	2°51′1″ E	38	Spunta	12
		Zeralda - Field 2				Spunta	13
		Staoueli	36°45′21″ N	2°53′25″ E	36	Spunta	14
	Boumerdès	Khemis El Khechna	36°38′56″ N	3°19′44″ E	77	Spunta	15
	Blida	Meftah - Field 1	36°37′0″ N	3°13′60″ E	180	Spunta	16
		Meftah - Field 2				Spunta	17
		Meftah - Field 3				Spunta	18
		Meftah - Field 4				Spunta	19
	Bouira	Aïn Bessem	36°17′48″ N	3°40′12″ E	675	Spunta	20
	Ain Defla	El Abadia	36°16′9″ N	1°41′4″ E	176	Spunta	21
		Ain Defla	36°15′55″ N	1°58′13″ E	273	Spunta	22
		Bourached	36°10′9″ N	1°55′45″ E	417	Spunta	31
West	Mostaganem	Sirat - Field 1	35°46′48″ N	0°11′31″ E	47	Spunta	2
		Sirat - Field 2				Spunta	3
		Fornaka	35°45′9″ N	0°1′1″ O	14	Spunta	4
		Aïn Nouissy	35°48′0″ N	0°3′0″ E	69	Desiree	5
		Hassi Mameche - Field 1	35°51′37″ N	0°4′23″ E	133	Spunta	6
		Hassi Mameche - Field 2				Spunta	9
		Mesra	35°50′14″ N	0°10′11″ E	79	Desiree	7
		Bouguirat	35°45′5″ N	0°15′12″ E	66	Spunta	8
	Chlef	Ouled Fares	36°13′58″ N	1°14′25″ E	136	Desiree	23
		Chlef	36°10′26″ N	1°20′12″ E	86	Desiree	24
	Mascara	Ghriss - Field 1	35°14′53″ N	0°9′41″ E	495	Desiree	25
		Ghriss - Field 2				Desiree	1
	Relizane	El Hamadna	35°54′0″ N	0°45′0″ E	79	Desiree	26
East	Tlemcen	Maghnia	34°51′42″ N	1°43′50″ O	495	Desiree	32
	Tébessa	Cheria	35°16′13″ N	7°45′7″ E	1090	Desiree	33
	Sétif	Guellal	36°2′42″ N	5°19′41″ E	911	Spunta	34
	Mila	Chelghoum Laid	36°10′0″ N	6°10′0″ E	922	Spunta	35
	Guelma	Bouchegouf	36°28′18″ N	7°43′47″ E	155	Spunta	36
South	El Oued	Hassi Khalifa - Field 1	33°36′4″ N	7°1′44″ E	35	Spunta	27
		Hassi Khalifa - Field 2				Spunta	30
		Trifaoui	33°25′24″ N	6°56′9″ E	68	Spunta	28
	Djelfa	Aïn El Ibel	34°21′17″ N	3°13′22″ E	1036	Spunta	29

## Data Availability

The data presented in this study are available in Table 1, Table 2, Table 3, Table 4, Table 5 and Table 6 and Figure 1, Figure 2, Figure 3, Figure 4 and Figure 5.
